# Genetic Analysis of Viruses Associated with Emergence of Rift Valley Fever in Saudi Arabia and Yemen, 2000-01

**DOI:** 10.3201/eid0812.020195

**Published:** 2002-12

**Authors:** Trevor Shoemaker, Carla Boulianne, Martin J. Vincent, Linda Pezzanite, Mohammed M. Al-Qahtani, Yagub Al-Mazrou, Ali S. Khan, Pierre E. Rollin, Robert Swanepoel, Thomas G. Ksiazek, Stuart T. Nichol

**Affiliations:** * Centers for Disease Control and Prevention, Atlanta, Georgia, USA; † Emory University, Atlanta, Georgia, USA; ‡ Ministry of Health, Riyadh, Saudi Arabia; § National Institute of Virology, Johannesburg, South Africa

## Abstract

The first confirmed Rift Valley fever outbreak outside Africa was reported in September 2000, in the Arabian Peninsula. As of February 2001, a total of 884 hospitalized patients were identified in Saudi Arabia, with 124 deaths. In Yemen, 1,087 cases occurred, with 121 deaths. Laboratory diagnosis of Rift Valley fever virus (RVFV) infections included virus genetic detection and characterization of clinical specimens by reverse transcription-polymerase chain reaction, in addition to serologic tests and virus isolation. Genetic analysis of selected regions of virus S, M, and L RNA genome segments indicated little genetic variation among the viruses associated with disease. The Saudi Arabia and Yemen viruses were almost identical to those associated with earlier RVF epidemics in East Africa. Analysis of S, M, and L RNA genome segment sequence differences showed similar phylogenetic relationships among these viruses, indicating that genetic reassortment did not play an important role in the emergence of this virus in the Arabian Peninsula. These results are consistent with the recent introduction of RVFV into the Arabian Peninsula from East Africa.

Rift Valley fever (RVF) (caused by Rift Valley fever virus [RVFV], family *Bunyaviridae*) is an emerging epidemic disease of humans and livestock, as well as an important endemic problem in sub-Saharan Africa. The virus is transmitted to livestock and humans by the bite of infected mosquitoes or exposure to tissues or blood of infected animals. Massive epizootics are typically observed in livestock during times of unusually high and sustained rainfall because of the presence of breeding sites and overabundance of adult competent mosquito vectors (1). Infections caused by RVFV are characterized by severe disease and abortion in livestock, particularly sheep and cattle. Persons in the epidemic region are at high risk for RVFV infection, potentially leading to thousands of human cases. Humans infected with RVFV typically have self-limited febrile illness, but retinal degeneration (5–10%), hemorrhagic fever (<1%), or encephalitis (<1%) may also develop (2).

We report the first confirmed outbreak of RVF outside Africa, in the Kingdoms of Saudi Arabia and Yemen. On September 10, 2000, the Ministry of Health in Saudi Arabia began to receive reports of unexplained hemorrhagic fever in humans near the Saudi-Yemeni border, with associated animal deaths and abortions. Patient samples from the outbreak were sent to the Centers for Disease Control and Prevention (CDC), where laboratory analysis confirmed the cases as being caused by RVFV. Genetic analysis was performed on all three viral RNA segments from human clinical samples, and the sequences were compared with previously characterized RVFV isolates to determine their genetic relatedness and geographic distribution.

## Materials and Methods

### Clinical Specimens

On September 15, 2000, acute-phase sera from four seriously ill, hospitalized patients with unexplained hemorrhagic fever were received by CDC for diagnostic assessment (Table 1). The shipment also contained sera from nine close contacts, mainly household members. A second shipment, which arrived on September 20, 2000, contained acute-phase sera from an additional 15 hospitalized patients and 12 contacts. Subsequent specimens from Saudi Arabia and Yemen were submitted for confirmation and more detailed analysis. All work with potentially infectious material was performed in a biosafety level 4 maximum containment facility.

**Table 1:** Results of diagnostic testing of initial Saudi Arabian Rift Valley fever outbreak specimens^a^

Patient ID^b^	Category^c^	ALK		C-CHFV			RVFV				
		IgM	IgG	Ag	IgM	IgG	Ag	IgM	IgG	ISOL	PCR
10901	Suspected case-patient	-	-	-	-	-	POS	-	-	POS	POS
10902	Suspected case-patient	-	-	-	-	-	POS	-	-	POS	POS
10904	Suspected case-patient	-	-	-	-	-	-	POS	POS	POS	POS
10905	Suspected case-patient	-	-	-	-	-	POS	-	-	POS	POS
10906	Contact	-	-	-	-	-	-	-	-	-	-
10907	Contact	-	-	-	-	-	-	-	-	-	-
10908	Contact	-	-	-	-	-	-	-	-	-	-
10909	Contact	-	-	-	-	-	-	-	-	-	-
10910	Contact	-	-	-	-	-	-	-	-	-	-
10911	Contact	-	-	-	-	-	-	-	-	POS	POS
10912	Contact	-	-	-	-	-	-	POS	-	-	-
10913	Contact	-	-	-	-	-	-	-	-	-	-
10914	Contact	-	-	-	-	-	-	-	-	-	-
10931	Suspected case-patient	-	-	-	-	-	POS	-	-	POS	POS
10933	Suspected case-patient	-	-	-	-	-	-	POS	POS	-	-
10935	Suspected case-patient	-	-	-	-	-	-	-	-	-	-
10937	Suspected case-patient	-	-	-	-	-	POS	POS	-	POS	POS
10939	Suspected case-patient	-	-	-	-	-	-	POS	-	-	-
10941	Suspected case-patient	-	-	-	-	-	-	-	-	-	-
10943	Suspected case-patient	-	-	-	-	-	POS	-	-	POS	POS
10945	Suspected case-patient	-	-	-	-	-	-	POS	-	POS	POS
10947	Suspected case-patient	-	-	-	-	-	POS	POS	-	POS	POS
10949	Suspected case-patient	-	-	-	-	-	-	POS	-	POS	POS
10951	Suspected case-patient	-	-	-	-	-	POS	-	-	POS	POS
10953	Suspected case-patient	-	-	-	-	-	POS	-	-	POS	POS
10955	Suspected case-patient	-	-	-	-	-	POS	POS	-	POS	-
10957	Suspected case-patient	-	-	-	-	-	POS	-	-	POS	POS
10959	Suspected case-patient	-	-	-	-	-	POS	-	-	POS	POS
10960	Contact	-	-	-	-	-	-	-	-	-	-
10961	Contact	-	-	-	-	-	-	-	-	-	-
10962	Contact	-	-	-	-	-	-	POS	-	-	-
10963	Contact	-	-	-	-	-	-	POS	-	-	-
10964	Contact	-	-	-	-	-	-	POS	POS	-	-
10965	Contact	-	-	-	-	-	-	-	-	-	-
10966	Contact	-	-	-	-	-	-	-	-	-	-
10967	Contact	-	-	-	-	-	-	-	-	-	-
10968	Contact	-	-	-	-	-	-	-	-	-	-
10969	Contact	-	-	-	-	-	-	-	-	-	-
10970	Contact	-	-	-	-	-	-	POS	-	-	-
10971	Contact	-	-	-	-	-	-	-	-	-	-

^a^RVFV, Rift Valley fever virus; ALK**,** Alkhurma virus**;** Ig, immunoglobulin; C-CHFV, Crimean-Congo hemorrhagic fever virus; ISOL, virus isolation; PCR, polymerase chain reaction; POS, positive results; -, negative results. ^b^Initial specimens were all from persons living in Jizan Province. ^c^Suspected case-patients were defined as described earlier (3).

### Virus Antigen, Ig M, and IgG Detection in Patient Sera

Patient sera were tested for the presence of RVFV, Crimean-Congo hemorrhagic fever virus (C-CHFV) antigen, or immunoglobulin (Ig) M or IgG antibodies reactive with these viruses and Alkhurma virus, a member of the tick-borne encephalitis (TBE) complex that was recently discovered in Saudi Arabia (4). RVFV and C-CHFV antigen-capture assays were performed in an enzyme-linked immunosorbent assay (ELISA) format essentially as described (5). The RVFV assay used polyclonal hyperimmune ascitic fluid raised against RVFV strain Zagazig 501 as the capture antibody and rabbit hyperimmune serum raised against RVFV Zagazig 501 as the detector antibody. The C-CHFV assay used a sheep hyperimmune serum raised against a South African C-CHFV strain as the detector antibody, and a mouse hyperimmune ascitic fluid raised against C-CHFV strain IbAr10200 as the capture antibodies (6). IgM antibody titers were determined by IgM antibody–capture ELISA, with RVFV, C-CHFV, or Alkhurma virus–infected cell slurry prepared as described (5). IgG antibody titers were determined by using RVFV, C-CHFV, and Alkhurma virus–infected cell antigens in an ELISA format as described (5).

### Virus Isolation and RNA Extraction

Viral RNA was obtained directly from patient blood or serum collected during the outbreak or from virus isolated from patient serum that was passaged once in Vero E6 cells. A virus stock was prepared by placing 100 µL of patient serum (200010901) onto a confluent monolayer of Vero E6 cells in a T-25 flask. After the virus was allowed to absorb for 1 h at 37°C, 6–7 mL of Dulbecco, modified Eagle medium supplemented with 5% fetal calf serum (FCS) and antibiotics, was added to the T-25 flask and allowed to incubate at 37°C, 5% CO_2_. Cell cultures were checked daily for cytopathic effect (CPE), and after approximately 75% CPE was observed, remaining cells were scraped off and combined with the supernatant. A low-speed centrifugation removed most debris, and the resulting supernatant was stored at -80°C. Some cells were retained to perform immunofluorescence (IFA) directed at RVFV to check for positive cultures. Two hundred microliters of passage 1 cell/supernatant was placed into 1 mL of TriPure (Roche, Indianapolis, IN) for RNA purification. Saudi Arabia sample 2003043 and Yemen sample 2001373 were prepared by placing 200 µL of blood or serum, respectively, directly into 1 mL of TriPure. RNA was extracted onto glass beads by using a RNAid kit (Bio101, Carlsbad, CA) according to a modified protocol (7).

### Indirect Immunofluorescence Assay and RT-PCR

Virus-infected cells were tested for RVFV antigens by indirect immunofluorescence assay essentially as described (5), except cells were incubated with anti-RVFV immune mouse ascitic fluid.

The nucleic acid sequences of the partial S, M, and L segments of RVFVs were amplified by using a one-step reverse transcriptase polymerase chain reaction (RT-PCR) (Promega Access kit, Madison, WI), according to manufacturer’s protocol. The primers NSn (5′- TATCATGGATTACTTTCC-3′) and NSc (5′- CCTTAACCTCTAATCAAC-3′) were used to amplify a 661-Nt region (excluding primer sequences) of the virus S segment region encoding the NSs protein (8). The primers RVFFORI (5′-GTCTTGCTTGAAAAGGGAAAA-3′) and RVFREVE (5′-CCTGACCCATTAGCATG-3′) were used to amplify a 708-Nt region (excluding primers) of the virus M segment region encoding the G2 protein. Primers Wag (5'-ATTCTTATTCCCGAATAT-3′) and Xg (5′-TTGTTTTGCCTATCCTAC-3′) were used to amplify a 176-Nt (excluding primers) region of the L segment (9). The primers RVFREVE together with primer RVFFORA (5′-TGCTACCAGACTCATTTGTC-3′) were used to amplify the initial diagnostic fragment of 186 Nt (excluding primers) of the virus M RNA genome segment region encoding the G2 protein.

Electrophoresis of amplified DNA products was done on a 1.7% agarose gel in Tris-acetate-EDTA buffer. Following staining with ethidium bromide, specific DNA bands were located by UV translumination, sliced from the gel, and purified by using Qiaquick spin columns (Qiagen, Valencia, CA). Dye terminator cycle sequencing reactions were performed by using ABI PRISM Dye Terminator Cycle Sequencing Ready Reaction Kits with AmpliTaq DNA Polymerase FS (Applied Biosystems, Foster City, CA). Reaction products were purified by using Centri-sep spin columns (Princeton Separations, Adelphia, NJ) and sequences determined with an ABI 377 automated DNA sequencer (Applied Biosystems). Output chromatograms were analyzed with Sequencher 3.0 software (Gene Codes Corp., Ann Arbor, MI). RVFV sequences were aligned with those of previously characterized RVFVs (10) by using the PILEUP program of the Wisconsin Package Version 10.2 (Genetics Computer Group, Inc., Madison, WI). Maximum likelihood phylogenetic analysis was carried out by using PAUP4.0b10 (Sinauer Associates Inc., Sunderland, MA).

## Results

### Initial RVFV Diagnosis

In early September 2000, the Ministry of Health of Saudi Arabia received reports of unexplained hemorrhagic fever cases in the southern Tehama (coastal plain) region of southwestern Saudi Arabia. Subsequently, reports were also obtained by the Yemen Ministry of Health of a similar disease in the adjoining Tehama region of Western Yemen. Initial specimens included acute-phase sera from four hospitalized patients with suspected cases and sera from nine contacts (mostly family members). Based on the available clinical information, the differential diagnostic included CK ABBREVS Rift Valley fever, Crimean-Congo hemorrhagic fever, and Tick-borne encephalitis-like viruses. The four serum samples from the suspected case-patients were tested by antigen-capture ELISA with RVFV- or C-CHFV-reactive antibodies; IgM-capture ELISA with RVFV, C-CHFV, or Alkhurma virus–infected cell lysate antigens; IgG ELISA with RVFV, C-CHVF, or Alkhurma virus–infected cell slurry antigen; virus isolation with Vero E6 cells; and RT-PCR assays for detection of RVFV, C-CHFV, or TBE-complex virus RNA. Evidence of RVFV infection was found in all four patients with suspected cases (Table 1). No evidence of C-CHFV or Alkhurma virus infection was found. Three of four acute-phase sera were positive by RVFV antigen-capture ELISA, and the single negative serum was positive for RVFV IgM and IgG, suggesting a later stage of infection in this case. All four sera were positive by RVFV RT-PCR assay and subsequently yielded infectious RVFV by culture on Vero E6 cells.

Of the nine sera from close contacts, two showed evidence of RVFV infection. One was RT-PCR positive and virus isolation positive, and the other was positive for RVFV IgM antibodies. A second shipment of specimens yielded similar results, again confirming that RVFV was responsible for the outbreak in Saudi Arabia. In this second shipment, 13 of 15 hospitalized suspected case-patients had evidence of RVFV infection. Four contacts of case-patients also showed evidence of RVFV infection.

The rapid RVFV RT-PCR assay appeared to be a useful complement to the RVFV antigen and IgM-capture ELISA tests for diagnosis of acute illness, as it detected virus RNA in 15 of 16 serum samples that were subsequently found to be RVFV positive. Overall correlation between the various RVFV diagnostic assays was good. Nucleotide sequence analysis of the 186-Nt (excluding primer regions) PCR products amplified from these initial specimens confirmed the virus identity as RVFV and showed no nucleotide differences between the viruses detected in these Saudi Arabian patients. In addition, no nucleotide differences were detected in this 186-Nt region relative to viruses detected in an earlier outbreak in East Africa in 1997 (data not shown) (11).

### Detailed Genetic Analysis

RNA extracted from three representative viruses was chosen for more detailed genetic analysis. These included RNA from RVFV isolate (strain 200010901) obtained from the first RVFV-infected case-patient to be laboratory confirmed and representing the early phase of the outbreak in Saudi Arabia. This isolate was obtained from a serum sample collected on September 13, 2000, from this case-patient (Table 1), who was infected in Jizan Province. RNA extracted from a serum sample collected late in the outbreak in Saudi Arabia was also included (Table 2). This serum sample was collected on November 22, 2000, from a case-patient infected in Asir Province. The third RNA sample was extracted from a blood sample obtained from a case-patient in Yemen. With these RNA samples, we hoped to detect any genetic variation in the RVFVs active during the early and late phases of the outbreak in Saudi Arabia and to evaluate whether the same virus strain was responsible for disease in Saudi Arabia and Yemen.

**Table 2:** Specimens chosen for more detailed virus genetic analysis, Saudi Arabia and Yemen

No.	Collection date	Location	Specimen type	Passage history^a^
200010901	Sept. 13, 2000	Jizan Province, Saudi Arabia	Virus isolate	P1
2001373	Nov. 22, 2000	Asir Province, Saudi Arabia	Serum (human)	NA
2003043	Oct. 28, 2000	Northwest Yemen	Blood (human)	NA

^a^P1, first passage; NA, not applicable

A single nucleotide difference was observed between each of the Saudi Arabia and Yemen virus S RNA genome segment fragments analyzed (601 nt). Similarly, no nucleotide differences were found between the Saudi Arabia 200010901 and Yemen 2003043 virus M RNA genome segment fragments we analyzed (510 nt), and these differed from the Saudi Arabia 2001373 virus fragment by only 1 nt. All three viruses were identical for the L RNA genome segment fragment we analyzed (129 nt). These data demonstrate that the viruses in the early and late stages of the RVFV outbreak in Saudi Arabia are virtually identical to one another and to the virus causing disease in Yemen.

The results of phylogenetic analysis of the nucleotide sequence differences among the S, M, and L RNA genome fragments of the Saudi Arabia and Yemen viruses and previously described RVFVs are shown (Figure). Earlier maximum likelihood analyses had separated RVFVs into three broad groups, which predominantly contained viruses from North Africa, West Africa, and East/Central Africa (10). All three RNA segment trees obtained here have the Saudi Arabia and Yemen viruses grouped with the East/Central Africa viruses. Specifically, the S, M, and L RNA genome segments of the Saudi Arabia and Yemen viruses are closely related to those of viruses previously detected in outbreaks in East Africa, as represented by the Kenya 1997 and Madagascar 1991 virus isolates (Figure, A, B, and C). The nucleotide changes in the S, M, and L RNA genome segment fragments observed among the closely related Saudi Arabia/Yemen viruses and the Kenya 1997 and Madagascar 1991 viruses are synonymous changes, resulting in no amino acid differences among these viruses.

**Figure:**
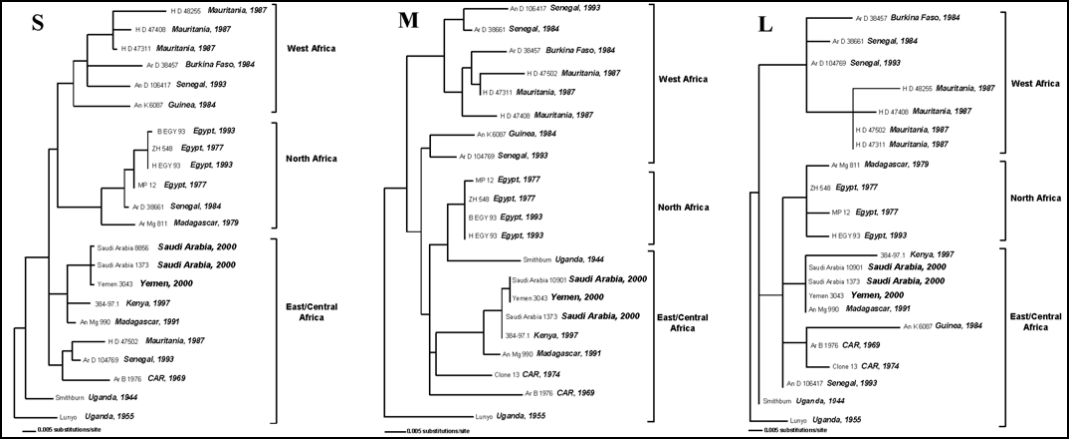
Phylogenetic relationship of the S, M, and L RNA segments of Rift Valley fever viruses. Maximum likelihood analysis of the nucleotide (nt) sequence differences among a 661-nt region of S RNA segment (Panel A), a 708-nt region of the M RNA segment (Panel B), and a 176-nt region of the L RNA segment (Panel C) of RVF viruses was performed by using PAUP4.0b10 (Sinauer Associates Inc., Sunderland, MA).

## Discussion

Using a combination of RVFV IgM and antigen-capture ELISA tests, along with the RT-PCR assay, we quickly identified RVFV as the cause of a large outbreak in Saudi Arabia reported in September 2000. The RT-PCR assay proved to be an excellent complement to the antigen and antibody ELISA detection systems for the initial rapid diagnosis of RVF. Virus-specific antibodies were present in three of four specimens that were positive by both virus isolation and PCR but negative by antigen capture, suggesting that immune complex formation (antibody blocking of antigen) may be the basis for the lower sensitivity of the antigen-capture assay. Although the IgM assay failed to identify 9 of 17 laboratory-confirmed (by virus isolation or PCR) acute RVF cases, the assay did detect recent RVFV infections in five contacts (mostly close family members) and one acute case that would have been missed on the basis of virus isolation or PCR assay only. The data from this study and others (12) demonstrate the importance of combining assays for the detection of virus (antigen capture, RT-PCR, or virus isolation) and the detection of virus-specific IgM to ensure that no acutely ill RVFV patients are missed.

This RVF outbreak, the first confirmed outside Africa, illustrates the potential for this disease to spread to other regions of the world. Virus activity on the Arabian Peninsula resulted in a considerable amount of disease activity from September 2000 to February 2001 (3,13). In Saudi Arabia, as of February 2001, 884 seriously ill, hospitalized RVF patients were identified, with 124 deaths. In Yemen, 1,087 cases were estimated to have occurred, with 121 deaths (14). The outbreak involved a broad geographic area, including Jizan and Asir Provinces in southwestern Saudi Arabia and much of the western coastal plain of Yemen. Because of the magnitude of this outbreak and the large geographic area it encompassed, the total number of human RVFV infections remains unknown. Data from previous outbreaks suggest that the number of hospitalized RVF patients identified during a large outbreak represents only a small percentage (<1%) of the total number of infections (2). Our finding of six laboratory-confirmed RVFV infections among household contacts is consistent with the view that hospitalized patients represent a small fraction of the number of infected persons. Based on these and earlier observations, the number of human infections during this epidemic must have been considerable. Large numbers of livestock were also affected, causing substantial losses and economic impact in the rural areas hardest hit by the disease. Further impact of the outbreak included trade and travel restrictions.

Genetic analysis of S, M, and L segments of the viruses detected in Saudi Arabia and Yemen indicated that essentially the same virus caused both outbreaks. Few genetic differences were detected between viruses sampled early and late in the outbreak or from the distant geographic regions of Jizan and Asir Provinces in Saudi Arabia and areas of Western Yemen. The lack of substantial genetic variation in these viruses, together with the lack of earlier disease reports, suggests that RVFV has only recently been introduced onto the Arabian Peninsula.

Phylogenetic comparison of the nucleotide sequence differences between the Arabian Peninsula RVFV S, M, and L segments and those of previously characterized RVFV isolates showed a close relationship between the Saudi Arabia/Yemen RVFVs and those circulating earlier in East Africa, particularly with the viruses responsible for the large RVF outbreak seen in the region in 1997–98 (11). These results are consistent with the introduction of RVFV into Saudi Arabia and Yemen from East Africa. While genetic reassortment has been observed in RVFVs associated with outbreaks in various geographic regions of Africa (10), the close phylogenetic relationship of the S, M, and L RNA segments of the 2000–01 Saudi Arabia and Yemen viruses and the earlier 1997 and 1991 Kenya and Madagascar viruses, respectively, provided no evidence of genetic reassortment among these viruses.

The mechanism of introduction of the virus into the Saudi Arabia and Yemen remains unknown. However, commercial trade of livestock is active from East Africa to the Arabian Peninsula, and disease is known to be endemic in the East African region. While no hospitalized RVF patients have been reported in 2002, whether this RVF lineage has become established in the Arabian Peninsula remains unclear. Surveillance of humans, livestock, and vector populations will continue to address this question.

## References

[1] Linthicum KJ, Anyamba A, Tucker CJ, Kelley PW, Myers MF, Peters CJ. Climate and satellite indicators to forecast Rift Valley fever epidemics in Kenya. Science. 1999;285:397–400. 10.1126/science.285.5426.397 10411500

[2] Meegan JM, Bailey CL. In: Monath TP, editor. The arboviruses: epidemiology and ecology. Boca Raton (FL): CRC Press; 1989.

[3] Centers for Disease Control and Prevention. Outbreak of Rift Valley fever—Saudi Arabia, August–October, 2000. MMWR Morb Mortal Wkly Rep. 2000;49:905–8. 11043643

[4] Charrel RN, Zaki AM, Attoui H, Fakeeh M, Billoir F, Yousef AI, Complete coding sequence of the Alkhurma virus, a tick-borne flavivirus causing severe hemorrhagic fever in humans in Saudi Arabia. Biochem Biophys Res Commun. 2001;287:455–61. 10.1006/bbrc.2001.5610 11554750

[5] Ksiazek TG, Rollin PE, Williams AJ, Bressler DS, Martin ML, Swanepoel R, Clinical virology of Ebola hemorrhagic fever (EHF): virus, virus antigen, and IgG and IgM antibody findings among EHF patients in Kikwit, Democratic Republic of the Congo, 1995. J Infect Dis. 1999;179:S177–87. 10.1086/514321 9988182

[6] Logan TM, Linthicum KJ, Moulton JR, Ksiazek TG. Antigen-capture enzyme-linked immunosorbent assay for detection and quantification of Crimean-Congo hemorrhagic fever virus in the tick, *Hyalomma truncatum.* J Virol Methods. 1993;42:33–44. 10.1016/0166-0934(93)90174-P 8320308

[7] Johnson AM, Bowen MD, Ksiazek TG, Williams RJ, Bryan RT, Mills JN, Laguna Negra virus associated with HPS in western Paraguay and Bolivia. Virology. 1997;238:115–27. 10.1006/viro.1997.8840 9375015

[8] Sall AA. de A Zanotto PM, Zeller HG, Digoutte JP, Thiongane Y, Bouloy M. Variability of the NS(S) protein among Rift Valley fever virus isolates. J Gen Virol. 1997;78:2853–8. 9367372 10.1099/0022-1317-78-11-2853

[9] Muller R, Poch O, Delarue M, Bishop DH, Bouloy M. Rift Valley fever virus L segment: correction of the sequence and possible functional role of newly identified regions conserved in RNA-dependent polymerases. J Gen Virol. 1994;75:1345–52. 10.1099/0022-1317-75-6-1345 7515937

[10] Sall AA, Zanotto PM, Sene OK, Zeller HG, Digoutte JP, Thiongane Y, Genetic reassortment of Rift Valley fever virus in nature. J Virol. 1999;73:8196–200. 10482570 10.1128/jvi.73.10.8196-8200.1999 PMC112837

[11] Sall AA. de A Zanotto PM, Vialat P, Sene OK, Bouloy M. Origin of 1997–98 Rift Valley fever outbreak in East Africa. Lancet. 1998;352:1596–7. 10.1016/S0140-6736(05)61043-4 9843109

[12] Sall AA, Thonnon J, Sene OK, Fall A, Ndiaye M, Baudez B, Single-tube and nested reverse transcriptase-polymerase chain reaction for detection of Rift Valley fever virus in human and animal sera. J Virol Methods. 2001;91:85–92. 10.1016/S0166-0934(00)00252-4 11164489

[13] Centers for Disease Control and Prevention. Update: outbreak of Rift Valley Fever—Saudi Arabia, August–November 2000. MMWR Morb Mortal Wkly Rep. 2000;49:982–5. 11098861

[14] Centers for Disease Control and Prevention. Outbreak of Rift Valley fever—Yemen, August–October 2000. MMWR Morb Mortal Wkly Rep. 2000;49:1065–6. 11186611

